# Building multi-sectoral alliances to co-design and pilot a gender-transformative comprehensive sexuality education intervention for adolescents: the case of *Si Yo Fuera Juan* in Uruguay

**DOI:** 10.1186/s12978-025-02257-x

**Published:** 2026-02-28

**Authors:** Alejandra López Gómez, Maria Lohan, Manuela Costa, María Soledad Ramos, Lía De Rosa, Sabrina Rossi, Pablo López

**Affiliations:** 1 Gender, Sexuality, and Reproductive Health Program at the Institute of Health Psychology, School of Psychology, Universidad de la República, Montevideo, Uruguay; 2 UNESCO Chair on Masculinities and Gender Equality | School of Nursing & Midwifery| Queen’s University Belfast| Belfast, Irlanda del Norte, Visiting Professor, HIAS, Hitotsubashi University, Tokyo, Japon

**Keywords:** Adolescents, Comprehensive sexuality education, Gender transformative approach, Rights based approach, Co-design intervention, Uruguay

## Abstract

**Background:**

Comprehensive sexuality education (CSE) is a fundamental adolescent right and key strategy for sexual and reproductive health and rights (SRHR). However, implementation remains uneven globally, particularly regarding gender-transformative CSE targeting boys. This study adapted, implemented, and evaluated “Si Yo Fuera Juan,” a gender-transformative CSE intervention for adolescents in Uruguay (2020–2023), highlighting two implementation strategies: (1) co-design with adolescents and multi-sectoral stakeholders, and (2) deliberate focus on male engagement.

**Methods:**

Using a rights-based approach, adolescents, teachers, parents, health professionals, policymakers, and civil society actors collaborated to culturally adapt, co-design and evaluate “Si Yo Fuera Juan.” The pilot intervention was implemented and evaluated in formal and non-formal educational settings across middle and low socioeconomic contexts in Uruguay. Mixed-methods data collection (virtual and in-person due to COVID-19) included focus groups and questionnaires with adolescents, workshops with families, and focus groups with teachers were conducted between 2020 and 2023. Qualitative data underwent deductive-inductive thematic analysis; quantitative data were analyzed descriptively by gender and socioeconomic status.

**Results:**

The intervention demonstrated high acceptability and feasibility across all stakeholder groups. Key findings included: strong support for the gender-transformative approach; identification of adolescents’, parents’, and teachers’ needs and recognition of gaps in current CSE provision; and the consensus that a cultural adaptation of the original intervention "If I Were Jack" was essential, requiring modifications to reflect Uruguayan social norms and institutional structures. Adolescents found the intervention emotionally engaging and realistic, facilitating critical reflection on gender roles and reproductive decision-making. Teachers valued the interdisciplinary approach and the innovative focus on boys. Implementation challenges included limited institutional time, insufficient teacher training, and infrastructural gaps.

**Conclusions:**

“Si Yo Fuera Juan” demonstrates successful adaptation of a gender-transformative CSE intervention through participatory co-design with multiple stakeholders. The intervention effectively engages boys in adolescent pregnancy prevention and has been integrated into national CSE policies in Uruguay. Sustained scaling requires institutional commitment, comprehensive teacher training, and strengthened intersectoral linkages between educational institutions and the health system.

**Plain English Summary:**

Comprehensive sexuality education (CSE) helps young people make informed decisions about their sexual and reproductive health. However, many CSE programs focus mainly on girls, leaving boys out of important conversations about pregnancy prevention and shared responsibility. This study describes how we adapted and tested "Si Yo Fuera Juan" (If I Were Juan), a sexuality education program designed to engage both boys and girls in Uruguay. Between 2020 and 2023, we worked with adolescents, teachers, parents, healthcare professionals, and government officials to create the adapted intervention to fit Uruguay's culture and educational system. The program uses an interactive video drama that shows realistic situations where young people face decisions about sexual and reproductive health and unintended pregnancy. This is combined with classroom activities that encourage discussion and critical thinking about gender roles and corresponsibility. We tested the program in schools and community settings with adolescents from different socioeconomic backgrounds. We collected information through surveys, focus groups, and workshops to understand what participants thought about the program. Results showed that adolescents, teachers, and parents found the program engaging and relevant. Young people appreciated the realistic scenarios and the safe space to discuss difficult topics. Teachers valued the focus on boys as an innovative approach to promote shared responsibility. However, teachers also noted challenges like limited time in school schedules and need for better training. The program has been incorporated into Uruguay's national sexuality education policies. To expand it successfully across the country, schools will need more support, including teacher training programs and better connections between schools and health services.

**Supplementary Information:**

The online version contains supplementary material available at 10.1186/s12978-025-02257-x.

## Background

Adolescent health and comprehensive sexuality education (CSE) are globally recognised as critical to the well-being and development of young people [1–4]. The sexual and reproductive health and rights (SRHR) of adolescents have profound implications—not only for their physical and mental health, but also for educational achievement, social and economic inclusion, and future family wellbeing [5, 6].

Since the 1994 International Conference on Population and Development (ICPD), international agreements and frameworks have advanced commitments to SRHR and the expansion of CSE. UNESCO defines CSE as a curriculum-based process that equips young people with knowledge, attitudes, and values to realise their rights, build respectful relationships, and make informed decisions about their sexuality. Effective CSE is rights-based, gender-transformative, participatory, and inclusive of diverse cultural perspectives [2, 7, 8].

Despite these policy advances, implementation of CSE remains limited and inconsistent across many countries. This is due to a combination of political, cultural, and structural barriers—including ideological resistance, religious and parental opposition, inadequate teacher training, and weak institutional support [7, 9, 10]. Research highlights that policy alone is insufficient: Implementation requires multi-level, intersectoral, multi-stakeholder strategies engaging national and local actors, and including boys as well as girls [8, 11–13]. When CSE programmes are embedded in broader systems of accountability and co-designed with youth and communities, they are more likely to gain legitimacy, overcome resistance, and lead to lasting change [14, 15].

Uruguay combines a strong democratic tradition with a secular welfare state shaped by early 20th-century reforms and sustained processes of social secularization. It is also a highly developed country, with a Human Development Index (HDI) of 0.83 and a global ranking of 52/193, and is classified by the World Bank as a high-income economy based on per-capita income. Within the 2030 Agenda, the country performs comparatively well in Latin America and the Caribbean (LAC). However, despite these favoring macroeconomic indicators and robust institutional structures, Uruguay continues to face deep structural gender inequalities in key areas of development. Its weakest performance relates to efforts to prevent and reduce gender-based violence (GBV): while femicide rates are the most visible and alarming indicator, GBV remains widespread and severely affects women, children, and adolescents [16].

This structural and policy context makes Uruguay a relevant case for examining gaps in the implementation of CSE. The country is a regional leader in SRHR policies, including access to abortion services based on defined legal grounds and regulated gestational limits [17, 18], as well as contraception and STI/HIV prevention and treatment [19]. Uruguay has ratified key human rights treaties and established mandatory CSE in schools [20, 21]. Since 2005, national policies have increasingly targeted adolescents’ SRHR, including efforts to integrate CSE into formal education [21, 22]. Nevertheless, implementation has been uneven, monitoring remains insufficient, and the meaningful participation of young people in policy development continues to be limited [22, 23].

Over the past decades, Uruguay has assumed multiple international commitments on human rights and more recently has advanced specific public policies aimed at reducing unintended adolescent pregnancy, supported by intersectoral coordination between health, education, and social-development agencies. Building on this framework and aligned with regional efforts to reduce and prevent unintended adolescent pregnancy, the government launched a comprehensive intersectoral strategy in 2016: the *National Intersectoral Strategy for the Prevention of Unintended Adolescent Pregnancy*, bringing together health, education, and social-development agencies, the University of the Republic, and UNFPA [24]; [25]. The strategy contributed to a significant decline in adolescent fertility, reaching a historic low in 2023: 20.9 births per 1,000 women aged 15–19, and 0.29 per 1,000 among girls under 15 [16]. These figures place Uruguay well below the regional average of 67 births per 1,000 women aged 15–19 in LAC [26].

Despite a rights-based legal framework, adolescent pregnancy persists in Uruguay due to socioeconomic and gender inequalities. Contributing factors include limited access to CSE, child sexual abuse and violence, GBV within adolescent intimate relationships, abortion stigma, and traditional gender norms that reinforce the maternal ideal [20, 27]. Traditionally, policy efforts to prevent adolescent pregnancy have focused exclusively on girls and young women [20, 25], obscuring the need for an active engagement of adolescent boys and young men. Gender-transformative interventions that engage men are strongly supported by international evidence, particularly for addressing issues such as unintended pregnancy—often framed solely as a women’s issue [28, 29] This approach is based on contemporary theories of gender and masculinities, which understand gender as a relational, socially constructed system embedded in power relations [30, 31]. From this perspective, dominant forms of masculinity—often associated with sexual entitlement, emotional restraint, and limited reproductive accountability—shape adolescents’ sexual behaviors and decision-making processes, reinforcing unequal burdens on girls and young women. Engaging boys and young men in CSE is therefore essential not only to promote individual behavioural change but also to contribute to broader gender-transformative shifts in norms, responsibility, and reproductive agency.

As part of the national intersectoral strategy, international evidence-based interventions engaging adolescent boys in pregnancy-prevention efforts were reviewed to assess their feasibility for adaptation in Uruguay. Between 2020 and 2023, an evaluation of the acceptability and pilot implementation of a culturally adapted version of *If I Were Jack*, a gender-transformative CSE intervention originally developed by a team at Queen’s University Belfast (QUB, Northern Ireland) [11, 32], was conducted. This programme uses an interactive video drama (IVD) that follows the story of Jack, a teenage boy facing an unintended pregnancy with his girlfriend Emma. The video includes a self-administered, individual, anonymous, and confidential questionnaire that unfolds as Jack and Emma’s story develops. Through group and individual classroom activities designed for inclusion in CSE programs with adolescents aged 14 to 16 years, students explore gender norms, responsibility, and decision-making. These activities enable teachers to work with adolescents and their families on a range of topics related to CSE, including decision-making, shared responsibility, consent, family planning, and gender stereotypes. A randomised controlled trial in the UK demonstrated that the programme increased contraceptive use, improved gender-equitable attitudes, and strengthened intentions to prevent unintended pregnancy among adolescents [11, 32, 33].

Although both Uruguay and Northern Ireland confront challenges related to adolescent SRHR, they are shaped by markedly different historical trajectories, cultural values, and institutional arrangements. While both contexts face difficulties in engaging adolescent boys in sexuality education and preventing unintended pregnancies, the sociocultural roots of these challenges differ. In Uruguay, resistance to CSE has emerged mainly from emerging contemporary conservative and anti-gender discourses within a predominantly secular welfare-state tradition [34, 35]. In contrast, in Northern Ireland, debates on sexuality, gender, and reproduction have been shaped by historical religious divides, community-based moral frameworks, and the legacy of political conflict [36]. These different cultural landscapes require differentiated strategies for the implementation and adaptation of gender-transformative CSE interventions.

This study analyses the acceptability, co-design and pilot implementation of “*Si yo fuera Juan”*, the Uruguayan adaptation of *If I Were Jack*, undertaken between 2020 and 2023 as part of a gender-transformative approach to CSE. It examines two innovative strategies developed during the cultural and contextual adaptation process: a multi-stakeholder collaboration engaging adolescents alongside teachers, parents, policymakers, and civil society actors in a co-design process [15, 28], and a focus on adolescent boys to promote corresponsibility in SRH decision making [29, 32, 33].

## Method

A Rights Based Approach (RBA) guided the study design [37]. This framework incorporates participatory approaches to ensure those most affected by research are included and emphasizes three requirements [38]: The goal must advance human rights; the process must follow human rights standards and principles; and the outcomes should strengthen both duty-bearers’ capacity to fulfill obligations and rights-holders’ capacity to claim their rights.

In order to incorporate a newly adapted CSE tool to the Uruguayan context a mixed methods approach was designed [39, 40], integrating qualitative methods (focus groups, interviews, and workshops) and quantitative methods (self-administered questionnaires). Qualitative methods enabled in-depth exploration of stakeholder perspectives and cultural adaptation needs through participatory processes, while quantitative methods allowed for evaluation of intervention acceptability and feasibility across a larger sample of adolescents, mitigating social desirability bias and peer influence effects and facilitating more honest disclosure on sensitive topics related to SRHR.

This study was conducted in three stages with sequential integration of methods: Stage one aimed to analyse the acceptability of the *If I Were Jack* CSE intervention to evaluate its potential adaptation to Uruguay’s curriculum. Stage two focused on the co-design of the intervention for Uruguay with key stakeholders. Finally, Stage three consisted of the pilot implementation and validation of the adapted intervention, “*Si yo fuera Juan*”. Qualitative methods were prioritized in Stages one and two to inform the adaptation process, while in Stage three both qualitative and quantitative methods were integrated to evaluate implementation.

This study was reported following the Good Reporting of A Mixed Methods Study (GRAMMS) framework [41]. See appendix N°1 for the detailed reporting model.

### Participants and recruitment

Inclusion criteria followed the RBA and multisectoral approach, identifying rights-holders and duty-bearers. Convenience and purposive sampling via snowball techniques through organizational networks and social media networks were used throughout stages one and two. At Stage three, the pilot intervention was developed selecting four public schools and three non-formal educational centres (youth community-based centers) using purposive sampling criteria. Schools were selected by the Gender and CSE Network of the National Public Education Administration (ANEP): two from middle-income neighborhoods and two from low-income areas. The youth community-based centers were selected in agreement with civil society organisations and located in low income neighbourhoods of Montevideo (the capital city).

The main stakeholders of this study were:

- * Adolescent Advisory Committee* (AAC): During all stages, we involved a group of adolescent boys and girls (under 18 years old) from different areas of the country. The recruitment and selection were made through the National Institute of Youth, ANEP and civil society organizations.
- *Expert Advisory Committee* (EAC): During all stages, we involved a group of experts on CSE and SRHR from academia, civil society organizations, UNFPA, UNESCO and Pan American Health Organization (PAHO).
- *Policymakers*: Throughout the process, at least one member from each of seven government agencies at both national and local levels that endorsed the project participated. At Stage two, we added the participation of educational specialists in CSE.
- *Teachers*: During Stage one and three, teachers were selected to participate from different backgrounds, ages and areas of the country, with experience in CSE, in agreement with the Gender and CSE Network of ANEP.
- *Health professionals*: During Stage one, health professionals of the National Health System, from different backgrounds, ages and areas of the country were selected to participate. Recruitment was conducted using snowball techniques and through social-media networks.
- *Parents*: During Stage one and three, parents from diverse socioeconomic contexts with children between 15 and 17 years-old participated. Recruitment was conducted through snowball techniques and social-media networks.
- *Adolescents*: On Stage one and three, adolescents aged 14–17 from diverse socioeconomic backgrounds and areas of the country were invited to participate. A call was made through social-media networks and civil society organizations for selection in Stage one. Adolescents from the selected schools and community-based youth centers were involved during Stage three of pilot implementation.

### Data collection

Data collection was conducted in Uruguay between May 2020 and December 2023, spanning the period of COVID-19 public health restrictions. This context required a combination of virtual and face-to-face research activities. Triangulated qualitative (interviews, focus groups and workshops) and quantitative methods (self-administered questionnaires) were used for data collection and analysis throughout all stages. 393 participants were recruited across 39 research activities in the three stages (see Table 1). See appendix N°2 for the questionnaire used at Stage one to evaluate acceptability of the initial *If I Were Jack* intervention and appendix N°3 for the questionnaire used to evaluate the “*Si Yo Fuera Juan”* version at Stage three.

**Table 1:** Participants and data collection of stages 1, 2 and 3

Stage	Data collection & activities	Participants	Number (*n*)
*Stage one: Analysis of acceptability and needs.*	4 Focus groups	Adolescents	39
	2 Focus groups	Health professionals	16
	4 Focus groups	Teachers	25
	2 Focus groups	Parents	19
	4 interviews	Policymakers	6
	1 workshop	Policymakers	10
	3 workshops	Expert Advisory Committee	16
	2 workshops	Adolescent Advisory Committee	10
*Subtotal*	*22*	*7*	141*
*Stage two: Co-design of the intervention for Uruguayan context*	6 interviews	Policymakers	6
	4 workshops	Adolescent Advisory Committee	5
	4 workshops	Expert Advisory Committee	16
	3 workshops	Teachers	10
	1 workshop	Adolescent Advisory Committee, Expert Advisory Committee	21
*Subtotal*	*18*	*3*	37*
*Stage three: Pilot intervention and evaluation*	1 evaluation questionnaire	Adolescents	109
	4 focus groups	Adolescents	69
	7 interviews	Teachers	15
	1 workshop	Teachers	10
	2 workshops	Parents	12
*Subtotal*	*15*	*3*	215(**)
***Total***	***39***	***7***	393(**)

(*) unique persons

(**) due to an anonymised questionnaire, it is not possible to establish the number of unique participants

#### Stage one: analysis of acceptability and needs

These activities took place between April 2020 and February 2021. We conducted 12 focus groups with adolescents (*n* = 39), parents (*n* = 19), teachers (*n* = 25), and health professionals (*n* = 16), along with four individual interviews and one workshop with CSE policymakers (*n* = 16 total), and five workshops with the EAC and AAC (*n* = 26) to assess: (a) perceptions of CSE needs in the Uruguayan context, and (b) perceptions of how the If I Were Jack intervention could address these needs. To facilitate the latter assessment, participants were given a demonstration of a translated version of the If I were Jack intervention.

Focus groups lasted approximately 60–90 min and interviews 30–45 min; all sessions were audio-recorded with participant consent and transcribed verbatim. Focus groups explored three main areas: (1) CSE context and needs: current provision, identified gaps, adolescent sexual and reproductive health needs, and access barriers; (2) family and institutional dynamics: communication patterns between families, educational institutions, and adolescents regarding sexuality education; and (3) intervention acceptability and adaptation: perspectives on the intervention’s content, format, and decision-making scenarios; views on gender roles, consent, and shared responsibility; and specific cultural adaptation needs for the Uruguayan context, including modifications to video narratives and classroom activities to align with local educational structures and sociocultural norms.

Parent focus groups were stratified by gender (separate groups for mothers and fathers) to explore potential differences in parental perspectives and communication patterns regarding adolescent sexuality education. Focus groups with other stakeholders (teachers, health professionals, and adolescents) were mixed-gender, as gender-specific separation was not considered relevant for those participant categories.

Due to the COVID-19 pandemic context, data collection was conducted on digital platforms, except for two focus groups with adolescents that were conducted face-to-face in communities with high adolescent fertility, low school participation, and vulnerable socioeconomic contexts. This decision sought to minimize selection bias related to access to digital technologies.

#### Stage two: Co-design of the intervention for Uruguayan context

The AAC and EAC were created in 2020 to lead the adaptation process. Between February 2021 and June 2022, we conducted six interviews with policymakers (*n* = 6), three workshops with teachers (*n* = 10), and nine workshops with the EAC and AAC (*n* = 21) to analyze Stage one results and develop the “*Si yo fuera Juan”* intervention, including the IVD, embedded questionnaire, teacher guidelines, and classroom materials. Workshops lasted 90–120 min and interviews 30–45 min; all sessions were audio-recorded with participant consent and transcribed verbatim. Workshops addressed specific adaptation needs identified in Stage one, including language localization, cultural contextualization of scenarios and characters, alignment with Uruguayan CSE curriculum requirements, and integration of national SRHR policies. Most activities took place on digital platforms. The “*Si yo fuera Juan”* intervention was validated prior to pilot implementation by the EAC, AAC, UN organizations and public agencies involved.

#### Stage three: pilot intervention and evaluation

The pilot implementation took place between July 2022 and December 2023. Teachers from participating schools and community centers were trained on the implementation, which was monitored by the research team. For the evaluation of the implementation, we combined four focus groups with adolescents (*n* = 69), a self-administered questionnaire completed by adolescents (*n* = 109), two workshops with parents (*n* = 12), and seven interviews and one workshop with teachers (*n* = 25 total). Focus groups and workshops lasted approximately 60–90 min and interviews 30–45 min; all sessions were audio-recorded with participant consent and transcribed verbatim. Focus groups and workshops in this stage were mixed-gender, as the evaluation focused on implementation experiences and feasibility rather than gender-specific perceptions. All data collection activities in this stage explored participants’ experiences with the intervention, perceived relevance and usefulness, suggestions for improvement, and feasibility of implementation in different educational contexts.

### Data analysis

Qualitative data through the different stages were managed using software Atlas.Ti v.7 and examined using a thematic analysis [42, 43], following a combined deductive–inductive approach: initial codes were informed by sensitizing concepts from the literature and interview guidelines and then refined inductively based on the collected data. The steps involved in this process were: (a) data were transcribed and formatted, (b) three researchers independently coded the data, (c) researchers analyzed inter-group difference, (d) researchers also analyzed intra-groups difference, especially related to gender and socioeconomic level.

Quantitative data from pilot evaluation questionnaires were analyzed using IBM SPSS v.24 and Google Colaboratory (Python IDE), with attention to socio-economic and gender differences, using descriptive statistics of Likert-scale items related to the intervention´s acceptability and feasibility.

Integration of methods occurred at two points in the study. First, qualitative findings from Stage one informed the co-design process in Stage two, where the AAC, EAC, teachers, policymakers and researchers used these insights to adapt the intervention’s content, language, and cultural elements to the Uruguayan context. Second, during Stage three evaluation, qualitative and quantitative data were analyzed separately and then integrated during the interpretation phase to provide a comprehensive assessment of the intervention’s acceptability, feasibility, and implementation challenges. This integration allowed for triangulation of findings, where quantitative ratings of acceptability were contextualized and explained by qualitative narratives of participants’ experiences.

### Ethics declarations

 Ethical approval was obtained from the Faculty of Psychology, Universidad de la República Research Ethics Research Committee in 2020 before commencing data collection. All participants provided informed consent to participate in the study. For adult participants (teachers, health professionals, parents, policymakers, and members of the Expert Advisory Committee), written informed consent was obtained prior to participation. For adolescent participants under 18 years old, a two-step consent process was followed: first, written informed consent was obtained from parents or legal guardians; second, written assent was obtained from the adolescents themselves. The Adolescent Advisory Committee (AAC) designed age-appropriate audiovisual consent materials to ensure adolescents fully understood the study’s purpose, procedures, potential risks, and benefits. All participants were informed of their right to withdraw from the study at any time without consequences.

## Results

### Stage one: analysis of acceptability and needs

During Stage one, we gathered detailed socio demographic data of the focus group participants. Most adult participants were women and had diverse educational and professional backgrounds: parents had high levels of formal education, health professionals included medical doctors, midwives, and mental health providers, and education professionals held both teaching and support roles. Adolescents, with a mean age of 16 years and identifying predominantly as female, but also as male and non-binary, came from diverse socioeconomic backgrounds. Over half reported a low socioeconomic status, and the majority were still attending secondary education. Sociodemographic data is detailed in Table Nº 2. (Table 2).

**Table 2:** Table participants demographic data

Group	*N*	Age (Mean)	Gender	Race/Ethnicity	Residence Area	Religious Affiliation	Education Profession	Socioeconomic Level
Parents	19	47.5	Female (52,6%), Male (47,3%)	White (89%), Other (11%)	Montevideo (61%), Rest of country (39%)	None (72%), Christian (28%)	Tertiary education (72%), Secondary education (28%)	Medium-high
Health professionals	16	40	Female (93,7%), Male (6,2%)	White (89%), Other (11%)	Montevideo (50%), Rest of country (50%)	None (81%), Christian (19%)	Medical doctors (44%), Mental health professionals (13%), Nursing and social care (13%), Midwives (38%)	No data
Teachers	25	44	Female (96,0%), Male (4,0%)	White (88%), Indigenous (4%), Asian (4%), Other (4%)	Montevideo (52%), Rest of country (48%)	None (64%), Christian (36%)	Teaching staff (48%), Student support roles (28%), Other roles (20%)	No data
Adolescents	36	16	Female (68,4%), Male (28,9%), Nonbinary (2,6%)	White (61%), Afro or Black (19%), Indigenous (3%), Asian (6%), No data (8%)	Montevideo (72%), Rest of country (28%)	None (75%), Christian (19%), Umbanda (3%), Jewish (3%)	Incomplete secondary education (75%), Incomplete primary (6%), No data (3%)	Low (53%), Medium (17%), Medium-high (31%)

The predominance of female participants across stakeholder groups, particularly among health professionals (93.7%) and teachers (96%), reflects the gender composition of these professional sectors in Uruguay, where women constitute the majority of the workforce in education and health care. This gender imbalance may also reflect broader patterns of engagement with CSE topics, where women tend to be more involved in discussions about adolescent health and education [44]. Among adolescents, the higher proportion of female participants (68.4%) may indicate greater willingness among adolescent girls to participate in discussions about sexuality and reproductive health, though mixed-gender groups were maintained to capture diverse perspectives.

**Fig. 1:**
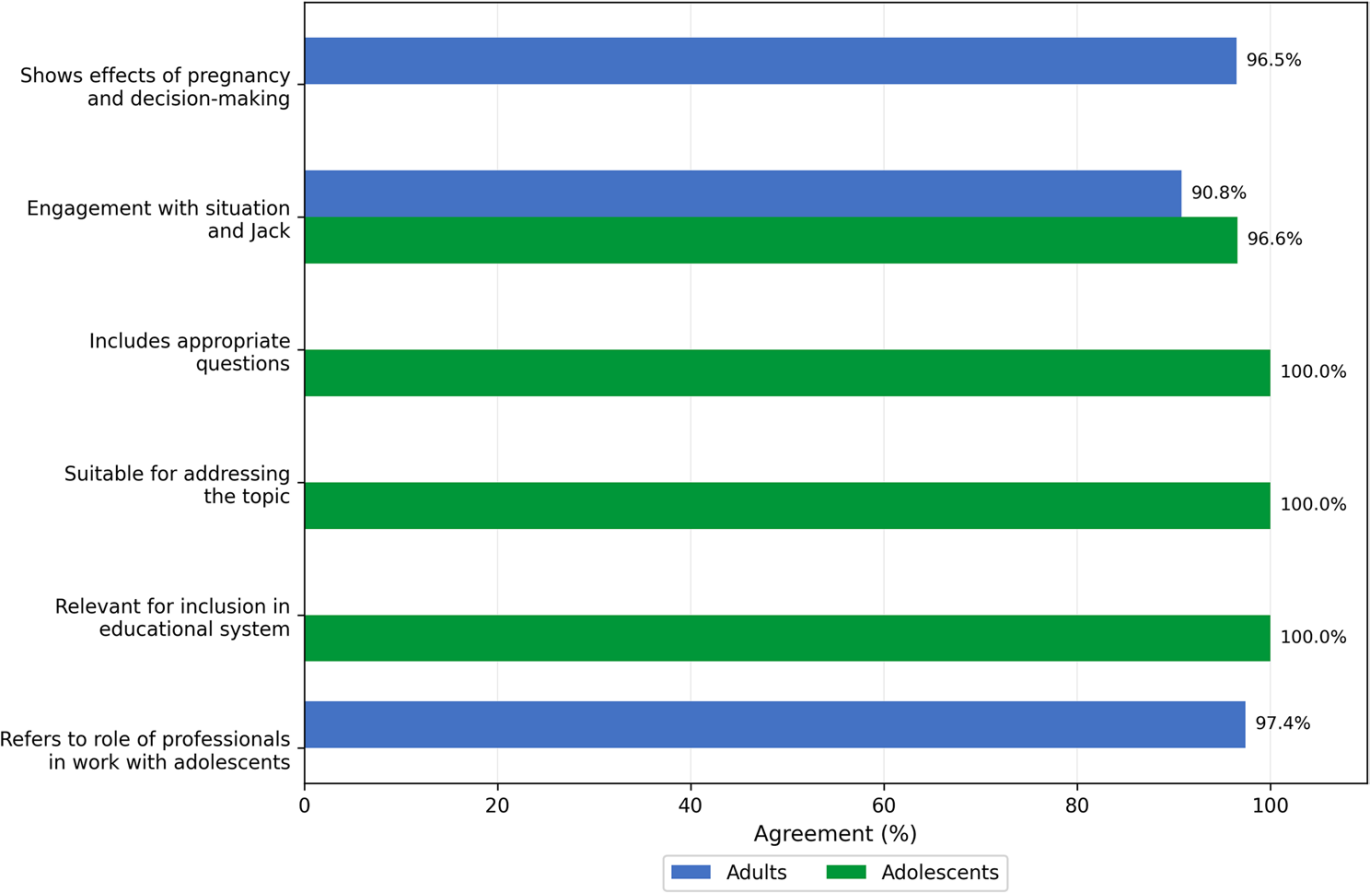
Participants attitudes regarding the If I were Jack intervention. Source: Questionnaire responded by adolescents and adults (*n* = 99)

Self-administered questionnaires distributed at the end of focus groups revealed high acceptability (> 90% agreement across all dimensions, Fig. 1 - see Appendix N°2 and N°3 for the questionnaire and appendix N°4 for questionnaire items), with strong consensus among adolescents and adults regarding adolescents’ capacity to engage with the issues and identify in the video.

Nonetheless, the acceptability was premised on the acknowledgement that a culturally adapted version should be created for use in Uruguay. Analysis of Stage one data revealed four key findings: (1) strong cross-stakeholder support for the intervention’s gender-transformative approach; (2) identification of adolescents’, parents’ and teachers’ needs and recognition of subsequent gaps in current CSE provision; and (3) consensus that cultural adaptation was essential for successful implementation, requiring modifications to reflect Uruguayan social norms and institutional structures.

#### Overall acceptability and gender-transformative approach

Findings confirmed the perceived need for culturally adapted CSE interventions and revealed positive attitudes towards adapting If I Were Jack to the Uruguayan context. The intervention was regarded as innovative due to its gender-transformative approach, interactive pedagogy, and digital components. The emphasis on involving adolescent boys in unintended pregnancy prevention was seen as a distinctive contribution, with policymakers viewing it as an opportunity to enhance the national CSE curriculum and mothers recognising it as addressing typically gendered responsibilities:

*“It is great that the proposal seeks to involve men in this situation*,* isn’t it? Because generally it is women who have to bear the burden of everything*,* from the moment they choose to have sex to using contraceptive methods”* (mother, focus group).

Adolescents appreciated the opportunity to challenge gender norms related to pregnancy prevention responsibilities, Some adolescents indicated that traditional gender norms are a barrier to male involvement in reproductive decision-making and that societal change is necessary to facilitate greater male participation. Adolescent girls were particularly surprised that the protagonist was male:

*“Usually*,* sex education is taught from the woman’s point of view*,* and… well*,* I had that thought and I was like — wow*,* that’s wild! I’m honestly surprised it’s not from the woman’s perspective. But I think it’s good — it shows how it affects men too*,* not just women.”* (Adolescent girl, focus group).

All participants highlighted the IVD’s ability to engage adolescents at both cognitive and emotional levels. The film was seen as appropriate for addressing unintended pregnancy, particularly due to its inclusion of multiple viewpoints—those of parents, peers, health services—and different outcomes (to continue the pregnancy -keep the baby or adoption- or choose abortion). Although the inclusion of adoption was somewhat controversial, participants agreed that it should remain part of the intervention to present a full range of possible decisions

#### Adolescents’, parents’ and teachers’ needs and barriers for CSE implementation

Parents reported feeling unprepared to address questions about sexuality and reproduction, indicating a strong need for guidance, support tools, and strategies to initiate and sustain conversations with their children. They viewed the intervention as a valuable tool to support family dialogue, stressing the importance of schools providing CSE in ways that engage families:

“*At what age do we start talking about this? And how do we approach it at different ages? (…) Personally*,* I feel like I’m missing a lot of tools.*” (mother, focus group).

Teachers identified several challenges to implementing CSE effectively. Key barriers included limited training on sexuality education topics, insufficient curriculum time to address CSE comprehensively, and concerns about potential resistance from families or school administration. They emphasized the need for structured materials, as well as institutional support and clear guidelines to navigate sensitive topics confidently.

*“There has to be the will on the part of the authorities to be able to put it into practice*,* to implement it.”* (teacher, focus group).

Adolescents criticized existing efforts for falling short in providing relevant information, fostering peer dialogue, and developing critical decision-making skills.

*“The sex education classes I had in high school were poorly organized — they lacked depth and information. That’s why I think this proposal would be great for high schools. It would offer a different perspective*,* provide more knowledge”* (adolescent boy, focus group).

Health professionals noted that adolescents, particularly boys, face significant barriers to accessing SRH services—including lack of information, bureaucratic obstacles, and unwelcoming health centers—underscoring the need for the intervention to not only educate adolescents but also improve their connection to existing services. Adolescents confirmed insufficient information about SRH facilities and unfriendly counseling approaches:

*“It’s hard to find a good place to get help here in Uruguay — there’s so much bureaucracy. (…) When a teenager goes to a health service*,* they start asking questions and you end up leaving without doing anything. You just shut down”* (adolescent boy, focus group).

#### Cultural adaptation of the intervention content and format

Qualitative data revealed several culturally specific elements that required careful modification of the intervention’s instruments. Adults and adolescents emphasized that certain narrative components of the IVD—particularly the emotional tone of the characters, the depiction of the health-care setting and counsellor, and the portrayal of family and peer dynamics—did not fully align with Uruguayan socio-cultural realities. For example, adolescents noted that the emotional responses of Jack and his partner appeared unusually restrained, which clashed with local expectations of how they would experience uncertainty, fear, or distress in the face of an unintended pregnancy. Similarly, the figure of the counsellor was perceived as unrealistic in the Uruguayan health system, where adolescents frequently encounter bureaucratic barriers and adult-centric practices.

*“Maybe if this were happening to us*,* we would have a totally different reaction. Maybe I would have a little more feelings (…) [Jack] hugs [Emma] really coldly.”* (adolescent, focus group).

*“I am doubting the accessibility of these spaces. It’s not something that happens overnight*,* as it appears in the video (…) when [adolescents] go to the health centre*,* they don’t even know where to go*,* they get turned away*,* maybe a month and a half goes by*,* and that’s when maybe they go with their mind already made up”* (health professional, focus group).

Modifications extended to visual and contextual dimensions of the IVD and embedded questionnaire. Participants emphasized the need for scenographic elements resonating with diverse Uruguayan audiences—neutral yet recognizable public spaces and socio-economic settings avoiding overrepresentation of advantaged contexts—as well as language adaptation incorporating colloquial expressions and culturally plausible response options. These modifications were understood as essential for strengthening identification, emotional engagement, and the perceived legitimacy of decision-making scenarios.

### Integration of quantitative and qualitative findings

High quantitative ratings of acceptability (over 90% agreement across all dimensions) were consistent with qualitative narratives from focus groups. The strong agreement on the intervention’s suitability for Uruguayan adolescents (Fig. 1) was reflected in adolescent girls’ expressions of surprise—*“wow*,* that’s wild! I’m honestly surprised it’s not from the woman’s perspective”*—, revealing not just acceptability but enthusiasm for the distinctive gender-transformative approach. While quantitative data showed strong agreement that adolescents could identify with the character of Jack, qualitative data revealed important nuances: adolescents appreciated the character’s emotional journey and decision-making process but emphasized the critical need for cultural adaptation to reflect Uruguayan language, social norms, and institutional contexts. This triangulation highlighted that acceptability was conditional—participants valued the intervention’s concept and pedagogical approach but insisted on substantial localization before implementation.

Both quantitative and qualitative data reveal consensus across all participant groups regarding the intervention’s potential value, though each stakeholder group emphasized different aspects: adolescents focused on peer relevance, realistic scenarios, and decision-making opportunities; parents emphasized family communication facilitation; teachers highlighted pedagogical structure and curriculum integration; health professionals stressed service connection and male engagement; and policymakers valued curriculum enhancement and innovation. This multi-stakeholder convergence strengthened the rationale for proceeding to the co-design phase while clearly identifying the adaptation priorities that would guide Stage two.

### Stage two: co-design of the intervention for Uruguayan context

Building upon Stage One findings of strong acceptability contingent upon cultural adaptation, Stage Two employed systematic co-design with policymakers, experts, adolescent advisors, and teachers to contextualise the intervention for Uruguay. This iterative process comprised 18 stakeholder interviews and workshops workshops with adolescents, the AAC, experts, the EAC, and teachers to develop the IVD, embedded questionnaire, implementation guidelines, and classroom materials. Through multi-stakeholder input, ‘If I Were Jack’ was systematically transformed into ‘Si yo fuera Juan’.

#### Cultural and linguistic localization of the IVD

The adaptation of the IVD from “If I Were Jack” to “Si yo fuera Juan” involved comprehensive modifications across multiple dimensions to ensure cultural relevance and resonance with Uruguayan adolescents. The AAC played a central role in shaping these adaptations, which addressed character development, language, setting, and accessibility.

Character development focused on reflecting how Uruguayan adolescents express emotions and relate to peers and adults. The protagonist was renamed “Juan”—a monosyllabic, culturally neutral name facilitating identification across socioeconomic contexts. A critical adaptation concerned emotional expressiveness: Stage One participants identified that the original characters appeared overly mature, formal, and emotionally restrained compared to typical Uruguayan adolescents. The adapted script incorporated more visible emotional struggle, with Juan showing greater anxiety and doubt throughout his decision-making process. To enhance authenticity, the script included informal nicknames between characters and references to contemporary adolescent activities (video games, school spaces).

Language localization proved critical for identification and engagement. The script was translated from English to Rioplatense Spanish, incorporating local expressions and colloquialisms characteristic of Uruguayan adolescents. The dialogue was adapted to sound natural and conversational, using expressions adolescents would actually use with peers and family. As one adolescent participant articulated:

*“If two Uruguayans did it*,* if they acted it*,* the way of speaking and everything would change a lot. It would be much easier to empathize*,* because things would be said much more as we are used to*,* with words we’re used to as well. Not so formal*,* but more like ‘pa*,* que viaje’ [wow*,* what a trip] or things like that that come naturally to you.”* (adolescent, focus group).

The adaptation carefully balanced authenticity with accessibility, selecting contemporary but not overly trendy slang to ensure the material would not quickly become dated.

The AAC recommended translating the IVD into Uruguayan Sign Language to ensure accessibility for deaf and hard-of-hearing adolescents, implemented through an on-screen interpreter in the final production.

Setting and visual elements were adapted to reflect recognizably Uruguayan spaces without being specific to particular neighbourhoods or social classes—including a park in the capital city, a typical neighbourhood, and a community football pitch. Cultural elements distinctive to Uruguay were incorporated to enhance local identification, including “mate” (the traditional Uruguayan infusion), deckchairs in public spaces, and contemporary adolescent cultural references. The visual aesthetic aimed for a contemporary but timeless quality, with clothing styles and physical environments that would feel current without quickly dating the material.

#### Scenario and content adaptations

A key point of discussion during co-design was the adaptation of the SRH counselling scene. In the original intervention, Jack and Emma visit a health centre where they meet with a professional counsellor in a dedicated, comfortable counselling space. Whilst there was consensus on the importance of making SRH services visible and modelling ideal care, participants acknowledged that the availability and quality of such services for adolescents vary widely across the country. Furthermore, the figure of a dedicated counsellor in a comfortable, informal setting was identified as uncommon in the Uruguayan health system, where adolescents frequently encounter bureaucratic barriers and adult-centric practices.

After deliberation, the research team and co-design participants adopted an aspirational but contextually feasible approach: portraying the counsellor as a professional working within the school setting, given that adolescents develop trust relationships with school-based professionals and school-based counselling would be more accessible and credible to adolescent viewers.

*“I was thinking about the educational centre as the place where the kids actually have someone closer to them*,* talking to kids who might be pregnant and talking to both of them*,* with the couple. I imagine [the counselling scene] closer to the educational centre.”* (EAC member, workshop).

The embedded self-administered questionnaire that adolescents complete as the story unfolds was adapted in several ways for the Uruguayan context. Participants indicated that questions were generally well-formulated but recommended “Uruguayanizing” both the language and response options to better reflect local realities. Policymakers recommended adapting alternatives to reflect Uruguayan legal and policy contexts, including abortion as a legalised and accessible option. Questionnaire data was stored in a secure online server to ensure confidentiality and ongoing collection of information.

Considering all these inputs from the co-design process, the Uruguayan version of the IVD was filmed between September and October 2021 by a local film production company, “La Penúltima Films,” starring Uruguayan amateur actors. A comprehensive summary of key film adaptations is provided in Appendix N°5.

#### Adaptation of classroom materials and implementation guidelines

To adapt implementation guidelines and classroom materials, workshops were held with experienced CSE teachers who provided input on activities, session structure, pedagogical approaches, and training requirements. Programme materials were published in 2023 [45], including a website (https://siyofuerajuan.uy/*)* with resources for parents, teachers, and adolescents. A comprehensive summary of key classroom materials is provided in Appendix N°6.

The intervention comprises an estimated nine hours organised into six 1.5-hour sessions, adapted to Uruguay’s education system whilst remaining flexible for community-based settings. Implementation guidelines include core and supplementary activities, a novel feature from the adaptation allowing centers to adapt the implementation format that best suited their context. Teachers valued the structured, ready-to-use format but stressed that flexibility was essential to integrate activities with existing content and emphasize topics relevant for specific groups.

Activities were co-designed for both formal settings (secondary schools, technical training) and non-formal settings (youth community centres) to maximize reach among socioeconomically vulnerable adolescents. Materials incorporated clear, step-by-step instructions with colour-coded thematic labels, enabling educators to identify key dimensions and prioritise according to adolescents’ needs. New activities were developed in response to teachers’ insights—for instance, a dedicated discussion space immediately after viewing the video to process initial reactions— and some activities were locally adapted reflecting both Uruguayan context and adolescent practices (such as adapting resources to local social media use).

Recognising Stage One findings regarding parents’ need for support, the intervention incorporates an at-home activity and family-oriented materials to facilitate dialogue on sexuality, pregnancy, and decision-making. Website resources include information on adolescent pregnancy, sexual violence, intimate partner violence, and online relationships, with policymakers contributing suggestions about the importance of including health service information. The at-home activity from the original If I Were Jack intervention was adapted to promote safe and constructive communication between adolescents and their families about sensitive issues, in line with recommendations emerging from teacher workshops. An additional optional family workshop was developed to strengthen school-family interaction, facilitated by designated teachers for parents or caregivers.

Comprehensive implementation guidelines were developed including session plans, facilitation strategies for sensitive discussions, CSE curriculum alignment information, and resources on SRH rights, services, and policies. Given teachers’ concerns about preparation, potential family opposition, and anti-gender discourse identified in Stage One, training addressed both content knowledge and confidence in facilitating challenging conversations.

#### Feasibility of implementation

Beyond adaptations to intervention content, the co-design process also assessed feasibility of implementation and identified enabling factors and potential barriers.

#### Enabling factors

The presence of an established CSE framework within the national education system was identified as an enabling factor for integrating the intervention into schools. The intervention was perceived as a valuable opportunity and a promising strategy to strengthen CSE in the country by adding value to the current curriculum as well as reinforcing and articulating the strategies implemented in non-formal educational settings [46].

Having multi-sectoral engagement from the outset was regarded as critical for implementation. Participants expressed their satisfaction with the co-design process and its outcome because it successfully incorporated the stakeholders’ perspectives, expectations and needs:

*“We highly value this project*,* which was developed through an intersectoral and multidisciplinary approach to address national needs. Our institution actively participated in adapting the materials in alignment with the secular principles upheld by ANEP. Through CSE interventions such as Si yo fuera Juan*,* the education system seeks to equip adolescents with the skills to co-construct life paths that allow them to fully exercise their citizenship and assume shared responsibility in all areas of life.* (policymaker, educational sector)

Active engagement of governmental partners through working meetings and collaborative discussions on cultural adaptation and implementation requirements enhanced institutional ownership and enabled integration of the intervention into national CSE policy for educational settings [47].

#### Barriers and challenges

However, participants noted weaknesses in the implementation of CSE, especially due to the limited allocation and insufficient training of human resources for this purpose. Questions remained about optimal curricular placement, with uncertainty about which specific courses or time slots would accommodate the intervention most effectively. The risk of CSE being relegated to “free hours” rather than integrated into core curriculum was a concern.

Teachers raised concerns about the growing influence of conservative anti-gender discourse, which positions CSE as a matter to be addressed exclusively within families. Some policymakers expressed concern about potential opposition from families in school-based CSE activities. This reluctance appeared to stem from a lack of confidence from teachers and limited experience in engaging with families on topics related to sexuality. To overcome this, teacher training and addressing communities were identified as crucial conditions.

*“It’s important to think about how we accompany and create protective communities for adolescents. (…) We have to break the logic of silence (…)*,* work in networks*,* creating a protection network within the community and beyond it. Si yo fuera Juan also came to strengthen and connect with that protection network”* (teacher, workshop).

**Fig. 2:**
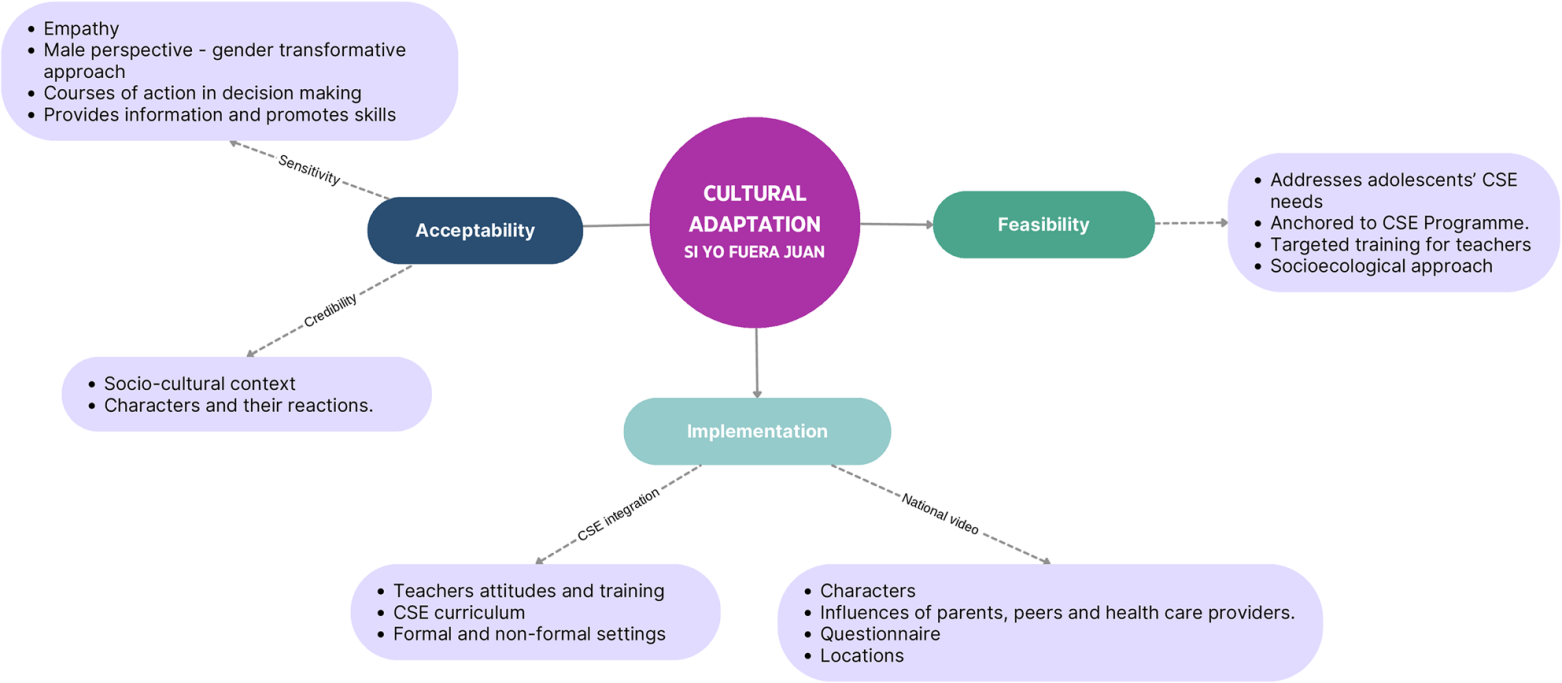
Cultural adaptation for Uruguayan context: From “If I were Jack” to “Si yo fuera Juan”

“Si yo fuera Juan” intervention was validated prior to pilot implementation by the Expert Advisory Committee, Adolescent Advisory Committee, UN organizations (UNFPA, UNESCO, PAHO), and public agencies involved in the project (ANEP, RAP-ASSE, INAU, among others), including several meetings with education authorities. This multi-stakeholder validation ensured that the adapted intervention met scientific, ethical, cultural, and policy standards. Figure 2 presents the three key components of the co-design and cultural adaptation process (acceptability, feasibility, and implementation) along with their main dimensions.

### Stage three. Pilot intervention and evaluation

For pilot evaluation, adolescents, teachers and parents from different educational settings highlighted positive aspects of the intervention and made suggestions regarding its implementation, identifying strengths as well as barriers. Findings reveal that strong intervention acceptability coexisted with significant implementation challenges. Adolescents valued the intervention’s pedagogical merit and praised its interactive format, privacy features, and male-centered perspective. However, substantial barriers emerged including technical difficulties, inconsistent pedagogical execution, limited curricular integration, insufficient teacher preparation, and minimal family participation. Triangulation demonstrated that implementation quality, rather than conceptual design, determined adolescent experiences, highlighting the need for systemic solutions addressing teacher training, infrastructure, and school-family partnerships.

### Intervention strengths

Pilot evaluation revealed strong acceptability across stakeholder groups, with adolescents, teachers, and parents identifying three core strengths that distinguished the intervention from conventional CSE approaches. First, the interactive format of the IVD enabled active decision-making rather than passive viewing, creating cognitive and emotional engagement that adolescents contrasted favourably with traditional educational methods. Second, the private response mechanism facilitated honest engagement with sensitive topics—particularly abortion and family dynamics—by eliminating peer judgment and creating emotional safety. Third, the thematic focus on unintended pregnancy proved relevant, with the male protagonist’s perspective valued as an innovative entry point for exploring shared reproductive responsibility.

*“I thought it was really good. The idea was that you could change things*,* you could give your own opinion on the story.”* (Adolescent girl, school).

*“It was good that all the decisions you made and the opinions you gave were private*,* that they stayed with you and no one could see your answer.”* (Adolescent boy, school).

The intervention’s ability to prompt personal reflection on beliefs, decision-making, and emotions regarding pregnancy represented a distinctive pedagogical contribution.

*“It made me think about what I would do if something like that happened to me.”* (Adolescent boy, school).

Teachers emphasised that the intervention functioned as a gateway for addressing broader SRHR themes—sexuality, gender, rights, and family dynamics—demonstrating adaptability across contexts with varying institutional priorities and student needs. This adaptability was seen as a strength, as well as a key for family involvement. However, teachers also identified the ready-to-use, structured materials as a major implementation facilitator, allowing them to focus on creating safe dialogue spaces rather than content development. In contexts characterised by limited preparation time and variable CSE training, this feature proved particularly valuable.

The interdisciplinary delivery model—where teaching teams from different disciplinary backgrounds collaborated—enriched discussions and supported pedagogical complementarity, benefiting both educators’ professional development and the quality of student engagement.

*“The guides were very clear and helpful. They provided a structure that made planning much easier.”* (Facilitator, youth centre).

*“I learned a lot from my colleague*,* especially things I didn’t know due to my limited training in sexuality education.”* (Teacher, school).

Teachers consistently emphasized that students responded positively to the activities, particularly those involving interactive or imaginative components, which fostered deep personal reflections, or those that enabled dialogue, encouraged open discussion, or disrupted routine classroom dynamics. This was confirmed by students.

*“I found all the activities very interesting and they provided good information (.) I liked all the ones that were discussed in the group”* (Adolescent boy, Youth Center).

Parents recognised the intervention as a meaningful opportunity to open dialogue and reflections on sensitive topics. Several parents reported that participation challenged their assumptions about their children’s perspectives and created opportunities for more open, supportive conversations—extending the intervention’s impact beyond the classroom setting.

*“the fact that [the intervention] allowed us to talk openly*,* that they could express themselves*,* and that it was brought home as well. What I realized was that this work was going to help him*,* but it also helped me a lot”* (mother, workshop).

*“I assumed that she thought the same way I did*,* but participating made me realize that her views were different from mine.”* (Mother, workshop).

### Implementation challenges and barriers

Despite strong acceptability, participants identified several implementation challenges requiring attention. Technical difficulties—particularly limited internet connectivity in some settings—highlighted the need for offline-accessible versions. Furthermore, adolescents described certain embedded questionnaire items as overly binary or lacking nuance, limiting their ability to express views authentically and suggesting the value of more varied response options and optional open-ended formats.

At the end of the IVD, the viewer does not find out what Juan (or his girlfriend) would choose regarding the pregnancy, in order not to infer one right or desirable answer. Some adolescents interpreted the open ending as a space for personal interpretation and reflection, while others expressed frustration at the lack of closure and the absence of visible consequences for Juan’s decisions. Suggestions included offering multiple possible endings depending on user choices, like other interactive formats.

*“I was left wondering—he never said anything at the end.”* (Adolescent boy, youth centre).

*“If they had shown a definite ending*,* it might have influenced us in some way.”* (Adolescent boy, school).

Several classroom activities were described by adolescents as repetitive or poorly followed up. Adolescents expressed a preference for more oral discussion, fewer mechanical or generic tasks, and more consistent engagement from teachers or facilitators throughout the process, which was not always the case during implementation.

*“They told us to do it and said they’d come get it the next day… and never came.”* (Adolescent girl, school).

Structural barriers emerged as more significant implementation constraints. Teachers described limited curricular alignment, with colleagues reluctant to allocate class time and providing only symbolic support. The intervention was frequently perceived as supplementary rather than integral to the pedagogical agenda, limiting its reach and potential for deep curricular integration.

*“It’s hard to mix this with the regular curriculum. Teachers say they’ll support it*,* but the actual support doesn’t happen.”* (Teacher, school).

Rapid implementation timelines hindered educators’ ability to prepare sessions thoroughly, affecting both their confidence in delivering content and their capacity to adapt materials to specific group needs. Teachers emphasised that successful delivery required broader training in sexuality education and rights beyond the intervention-specific preparation provided, underscoring the necessity for curricular and continuous training of educational staff as a central condition for the implementation of this type of CSE strategy.

*“Not every teacher can deliver this. You need training in sexuality*,* group management*,* and to know the law”* (teacher, school).

Family participation remained limited in both reach and depth. Parents reported uncertainty about how, when, and what to discuss regarding SRHR with their children, often either not completing the home-based activity or engaging superficially. This pattern—consistent with international CSE evidence—highlights persistent barriers including competing responsibilities, discomfort with sexuality topics, and insufficient guidance on facilitating conversations.

*“The first time they asked me what sex was*,* I would answer ‘female sex and male sex.’ That’s how I avoided the topic at all costs.”* (Mother, workshop).

#### Integration of quantitative and qualitative findings

**Fig. 3:**
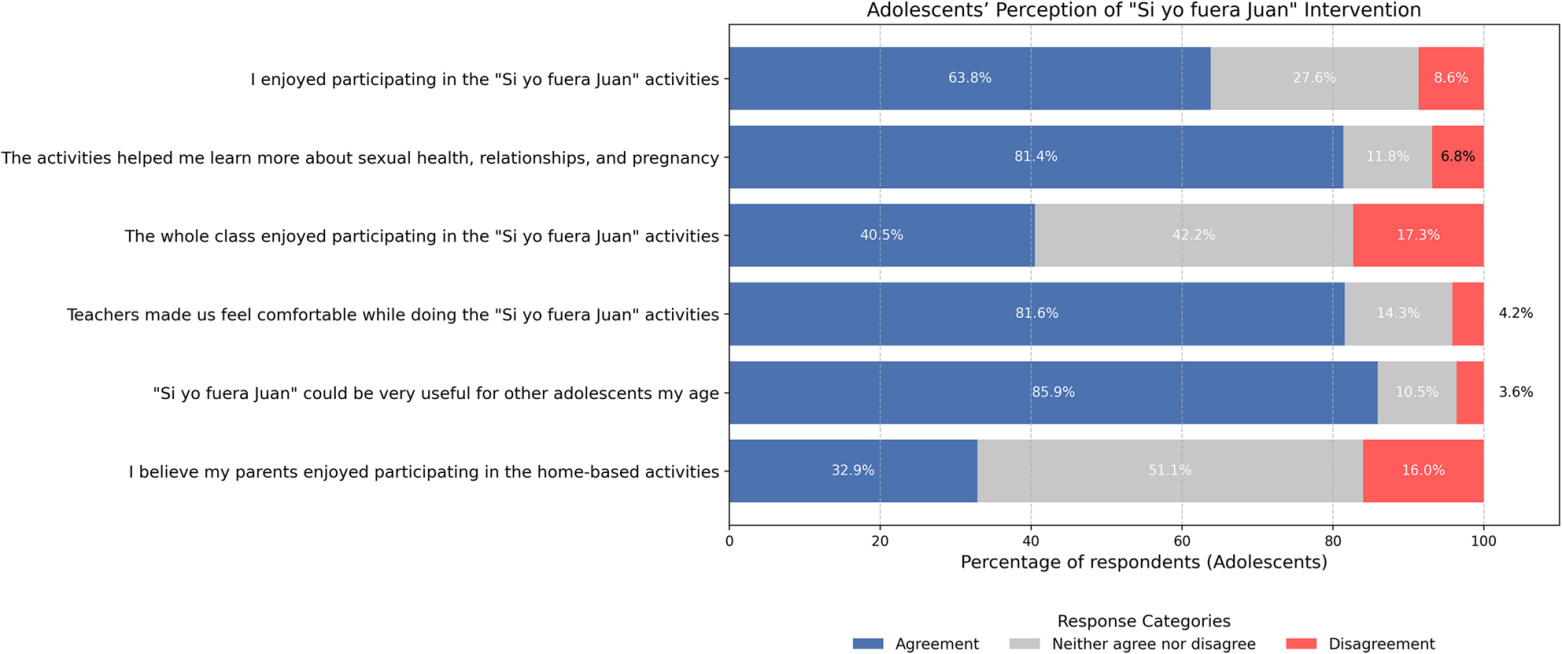
Adolescents Perception of “Si yo fuera Juan” intervention. Source: Questionnaire responded by adolescents (*n* = 109)

 At the end of the intervention, 109 adolescents completed an anonymous questionnaire (see Appendix N°3) to evaluate the implementation process, the quality of the activities, and the overall programme. Figure 3 summarizes the levels of agreement and disagreement regarding key aspects of the pilot implementation, including overall satisfaction with the content, the perceived quality of the information provided, and its potential value for other adolescents. Lower levels of agreement were observed in items related to perceptions of whether their peers enjoyed the activities and whether their parents or caregivers engaged in the home-based component.

Triangulation revealed three convergent patterns across methods. First, the gap between recommendation rates (85,9%) and personal enjoyment (63,8%) suggests adolescents distinguished pedagogical merit from experiential satisfaction—valuing the intervention’s educational importance despite mixed activity experiences. Qualitative accounts of repetitive tasks and inconsistent teacher follow-up explain why strong cognitive engagement (81,4% reported the activities helped them learn more about SRHR) coexisted with tempered satisfaction. This pattern underscores that implementation quality, rather than conceptual design, mediated adolescent experiences.

Second, consistently low scores for parental (32,9%) and peer (40,5%) enjoyment—compared to personal enjoyment (63,8%)—reveal adolescents’ limited capacity to assess others’ experiences. Beyond reflecting actual family barriers identified qualitatively, this pattern likely indicates insufficient communication about the intervention within families and peer groups. The multi-method convergence on limited parental participation (32,9% of adolescents agree on parent enjoyment) highlights not merely individual family deficits but systemic gaps in school-family partnerships. Effective CSE implementation requires whole-community approaches that strengthen institutional mechanisms for family engagement rather than relying solely on individual parent initiative.

Third, challenges identified by qualitative data—connectivity issues, rushed timelines—contextualised the 15–20% disagreement across items, reflecting variable implementation fidelity across sites where teacher training and institutional support differed. High ratings for teacher support (81,6% felt comfortable with activities) and information quality (81,4%) converged with qualitative narratives emphasising that trained, sensitised educators generated better implementation experiences.

Data integration reveals that whilst the intervention’s conceptual framework achieved strong acceptance, translating acceptability into consistent positive experiences requires addressing structural barriers: curricular integration, sustained teacher training, technological infrastructure, and robust school-family-community partnerships.

## Discussion

This study reported the adaptation and pilot implementation of “*Si yo fuera Juan”*, a culturally and contextually relevant version for Uruguay of the gender-transformative CSE intervention *If I Were Jack* implemented in the UK. The project applied two key innovations: youth co-design with diverse multi-sectoral stakeholders, and a deliberate focus on male engagement in SRHR. This responds to ongoing gaps not only for the country but also for Latin America and the Caribbean, where adolescent fertility remains high [48] and most policies focus exclusively on girls [49]. By centering adolescent boys alongside girls and fostering intersectoral collaboration, the intervention offers a promising model for more equitable and effective implementation of sexuality education [50].

Findings demonstrate high acceptability among all participants and support the intervention’s feasibility within the public education system in both school and non-formal settings. The overwhelmingly favorable reception suggests that the format—an IVD combined with classroom activities—was not only well received but also aligned with core CSE principles of cultural relevance, emotional engagement, and participatory learning [28].

A key strength of the initiative lies in its rights-based methodology. The co-design process actively involved adolescents, teachers, parents, health professionals, and decision-makers, ensuring that materials were culturally appropriate, pedagogically sound, and aligned with secular educational principles. This inclusive approach enhanced stakeholder ownership and contributed to the legitimacy of the intervention among the public institutions involved.

Adolescents particularly valued the IVD, describing it as emotionally engaging, realistic, and thought-provoking. They appreciated the opportunity to reflect on male roles and responsibilities in unintended pregnancy scenarios, as well as the safe and non-judgmental environment created by the intervention. While parents expressed interest in the intervention as an opportunity to build confidence in dialoguing with their children about CSE, their participation in pilot activities remained low. This pattern aligns with evidence from other contexts [51–53] and underscores the need for dedicated family engagement strategies that address barriers to participation.

Teachers praised the quality of the materials and the interdisciplinary framing of the proposal, highlighting the focus on boys as an innovative strategy to promote co-responsibility as a gender transformative strategy—one they believed could constructively influence other areas of CSE related to gender roles. Moreover, incorporating a focus on boys’ sexual and reproductive health, as well as promoting their responsibility in areas traditionally assigned to women—such as health care, sexuality education, and reproductive health—is not only emphasized in global and regional literature as a key component of CSE [54–57], but was also identified locally as a gap to be addressed [20].

Strengthening adolescents’ decision-making capacities emerged as a relevant and innovative aspect of the intervention, particularly in relation to how it addressed participants’ attitudes toward pregnancy, abortion, and adoption. This is especially important given previous literature indicating that adolescents’ attitudes toward available options influence their decision-making processes when facing an unintended pregnancy [58–62]. Building these capacities appears to be a crucial component to include in CSE interventions to ensure adolescents can fully exercise their sexual and reproductive rights and make informed, consensual decisions [63].

Despite the overall positive appraisal, the scalability and sustainability of the intervention remain to be fully evaluated in Uruguay and face important structural challenges. Several teachers highlighted limitations in the implementation of this CSE intervention, such as insufficient institutional time, lack of dedicated training in sexuality education, technological challenges in some schools, and the dependence on individual commitment to carry out activities. These issues reveal the fragility of implementation efforts when they rely solely on the initiative of motivated staff, rather than on systemic support mechanisms [63, 64].

Moreover, the persistence of conservative discourses in some institutional settings—often manifesting as reluctance or inaction—adds complexity to scaling up. Institutional hesitations underscore the subtle ways in which anti-gender discourse can influence implementation—namely through passive resistance, bureaucratic inertia, or a lack of family approaches from schools, as documented in previous regional literature [34, 35, 65–67]. For the intervention to be effectively expanded, it is essential to strengthen institutional frameworks, ensure ongoing training, and secure political and administrative backing that integrates CSE as a structural component of educational policy.

This study has methodological strengths, in particular the use of a combined qualitative and quantitative methods and the inclusion of diverse actors across design and evaluation phases. This methodological approach revealed important complementarities: qualitative data helped to explain the mechanisms behind quantitative acceptability scores, while quantitative data reinforced the significance of themes identified qualitatively, such as the centrality of gender norms in decision-making. At the same time, some limitations should be acknowledged. The sample was non-probabilistic and limited to specific regions of the country, which may affect the generalizability of findings. Survey measures alone could not capture the nuances of pedagogical implementation, and qualitative findings may overrepresent institutions already predisposed to support CSE. Additionally, the pilot evaluation focused on short-term acceptability and perceptions, without assessing long-term behavioural or attitudinal change.

Scaling “*Si yo fuera Juan*” nationally requires strengthening institutional capacity across multiple domains. First, teacher training remains central. In response to educators’ expressed needs, a hybrid professional development programme combining synchronous and asynchronous online components was developed. Institutionalizing and expanding this model would support consistent implementation across diverse educational settings whilst addressing the training gaps identified in this study.

Second, implementation must remain flexible and responsive to territorial and sociocultural variation. Local adaptation processes involving community actors are essential to ensure relevance in rural areas and peripheral urban neighbourhoods, where contextual realities may differ substantially from urban centres.

Third, dedicated family engagement mechanisms are needed. Stage Three findings revealed that parental participation remained limited despite the intervention’s potential to facilitate family dialogue. Establishing structured spaces for parent education and guidance—beyond optional workshops—may address the discomfort, information gaps, and communication challenges identified across stakeholder groups.

Fourth, stronger institutional linkages between education and health systems are required. Formal referral pathways and service integration can reinforce adolescents’ access to SRHR counselling and care, extending the intervention’s impact beyond classroom settings and addressing the healthcare access barriers documented in Stage One.

Finally, sustainable scaling demands policy-level integration: incorporating “*Si yo fuera Juan*” into national CSE frameworks, securing dedicated funding streams independent of project cycles, and establishing monitoring mechanisms that support rather than burden educators. The multi-sectoral alliances built through this study’s participatory design approach enabled public institutions to incorporate the intervention into their policy agendas—demonstrating that institutional ownership, not individual initiative, is the foundation for sustained implementation at scale.

## Conclusions

“Si Yo Fuera Juan” demonstrates that gender-transformative CSE interventions can be successfully adapted to Uruguayan context through participatory co-design. Strategic alliances with governmental partners, international agencies, and civil society—combined with direct consultation with adolescents, health professionals, teachers, and families—enabled the development of culturally relevant intervention addressing a critical gap: the engagement of boys and young men in adolescent pregnancy prevention alongside girls. The pilot study confirms high acceptability across stakeholder groups, feasibility within diverse educational settings, and potential to foster shared responsibility in sexual and reproductive health decision-making. This collaborative approach has facilitated integration of the intervention into national CSE and adolescent pregnancy-prevention policies currently being implemented in Uruguay.

## Supplementary Information

Supplementary Material 1.

Supplementary Material 2.

## Data Availability

The datasets analyzed during the current study are available from the corresponding author on reasonable request.
