# Supplementary material for: Building multi-sectoral alliances to co-design and pilot a gender-transformative comprehensive sexuality education intervention for adolescents: the case of Si Yo Fuera Juan in Uruguay
Source: Reprod Health. 2026 Feb 28;23:78. doi: 10.1186/s12978-025-02257-x (PMC13072552; doi:10.1186/s12978-025-02257-x)
Supplement: Supplementary file 1 — Supplementary Material 1. [file 12978_2025_2257_MOESM1_ESM.docx]

# Appendix 1, Questionnaire to evaluate acceptability of the If I Were Jack intervention.

**Parents**

It helped me understand the impact that an unplanned pregnancy would have on a teenager

☐ Strongly agree  ☐ Agree  ☐ Not sure  ☐ Disagree  ☐ Strongly disagree

It made me think about issues I hadn't considered before

☐ Strongly agree  ☐ Agree  ☐ Not sure  ☐ Disagree  ☐ Strongly disagree

It involved me in Jack's and his parents’ situation

☐ Strongly agree  ☐ Agree  ☐ Not sure  ☐ Disagree  ☐ Strongly disagree

It made me think that I should talk more with my children about this situation

☐ Strongly agree  ☐ Agree  ☐ Not sure  ☐ Disagree  ☐ Strongly disagree

It made me reflect on the importance of including these topics in the school curriculum

☐ Strongly agree  ☐ Agree  ☐ Not sure  ☐ Disagree  ☐ Strongly disagree

**Adolescents**

The video helped me put myself in Jack’s shoes

☐ Strongly agree  ☐ Agree  ☐ Not sure  ☐ Disagree  ☐ Strongly disagree

The questions helped me reflect on the topic presented in the video

☐ Strongly agree  ☐ Agree  ☐ Not sure  ☐ Disagree  ☐ Strongly disagree

I think it is an appropriate way to reflect and/or talk with others about the topic

☐ Strongly agree  ☐ Agree  ☐ Not sure  ☐ Disagree  ☐ Strongly disagree

I believe it can be useful for other teenagers to reflect and/or talk about the topic

☐ Strongly agree  ☐ Agree  ☐ Not sure  ☐ Disagree  ☐ Strongly disagree

It is good material to include in sex education in high schools and vocational schools

☐ Strongly agree  ☐ Agree  ☐ Not sure  ☐ Disagree  ☐ Strongly disagree

**Health professionals**

It made me reflect on my work with teenagers

☐ Strongly agree  ☐ Agree  ☐ Not sure  ☐ Disagree  ☐ Strongly disagree

It helped me understand adolescents’ decision-making regarding pregnancy

☐ Strongly agree  ☐ Agree  ☐ Not sure  ☐ Disagree  ☐ Strongly disagree

It made me think about how to approach this topic with teenagers

☐ Strongly agree  ☐ Agree  ☐ Not sure  ☐ Disagree  ☐ Strongly disagree

It involved me in Jack’s situation

☐ Strongly agree  ☐ Agree  ☐ Not sure  ☐ Disagree  ☐ Strongly disagree

I identified with the health professional in the video

☐ Strongly agree  ☐ Agree  ☐ Not sure  ☐ Disagree  ☐ Strongly disagree

**Teachers**

It made me reflect on my work with teenagers

☐ Strongly agree  ☐ Agree  ☐ Not sure  ☐ Disagree  ☐ Strongly disagree

It helped me understand adolescents’ decision-making regarding pregnancy

☐ Strongly agree  ☐ Agree  ☐ Not sure  ☐ Disagree  ☐ Strongly disagree

It made me think about how to approach this topic with teenagers

☐ Strongly agree  ☐ Agree  ☐ Not sure  ☐ Disagree  ☐ Strongly disagree

It involved me in Jack’s situation

☐ Strongly agree  ☐ Agree  ☐ Not sure  ☐ Disagree  ☐ Strongly disagree

I think it is a suitable tool to include in the sex education curriculum with teenagers

☐ Strongly agree  ☐ Agree  ☐ Not sure  ☐ Disagree  ☐ Strongly disagree

# Appendix 2, Questionnaire to evaluate Si yo fuera Juan intervention for adolescents.

1. What is your gender identity?

☐ Female  ☐ Male  ☐ Gender fluid  ☐ Other

2. How often were the “If I Were Juan” activities carried out in your school?

☐ Once a week  ☐ Twice a week  ☐ Other (please specify)

3. How would you rate the presentation of “If I Were Juan” carried out by the teachers at your school?

☐ Very goog  ☐ Good  ☐ Neither good nor bad  ☐ Bad  ☐ Very bad

4. What did you think about the video about Juan and Ema? (Open-ended)

5. Of all the activities done in class as part of “Si yo fuera Juan”, which one did you like the most, and why? (Open-ended)

6. Of all the activities done in class as part of “Si yo fuera Juan” , which one did you like the least, and why? (Open-ended)

7. Were you able to participate in all the activities?

☐ Yes  ☐ No

8. Did your parent, guardian, or family member participate in the home-based activity proposed by “Si yo fuera Juan.”?

☐ Yes  ☐ No

9. I enjoyed participating in the activities of “Si yo fuera Juan.”

☐ Strongly agree  ☐ Agree  ☐ Not sure  ☐ Disagree  ☐ Strongly disagree

10. The activities of “If I Were Juan” helped me learn more about sexual health, relationships, and pregnancy.

☐ Strongly agree  ☐ Agree  ☐ Not sure  ☐ Disagree  ☐ Strongly disagree

11. The whole class enjoyed participating in the activities of “Si yo fuera Juan.”

☐ Strongly agree  ☐ Agree  ☐ Not sure  ☐ Disagree  ☐ Strongly disagree

12. The teachers made us feel comfortable while doing the activities of “Si yo fuera Juan.”

☐ Strongly agree  ☐ Agree  ☐ Not sure  ☐ Disagree  ☐ Strongly disagree

13. I think “If I Were Juan” could be very useful for other teenagers my age.

☐ Strongly agree  ☐ Agree  ☐ Not sure  ☐ Disagree  ☐ Strongly disagree

14. I believe my parents (mother, father, or other adult family member) enjoyed participating in the home-based activities proposed by “Si yo fuera Juan.”

☐ Strongly agree  ☐ Agree  ☐ Not sure  ☐ Disagree  ☐ Strongly disagree

15. If you had to rate the “Si yo fuera Juan” program from 1 to 10 (where 1 is very bad and 10 is excellent), what score would you give it? (Rating from 1 to 10)

16. Do you have any suggestions to improve the “Si yo fuera Juan” program? (Open-ended)

# Appendix 3. Dimensions linked to questionnaire’ items

| **Dimension name** | **Items** |
| --- | --- |
| Shows the effects of pregnancy and decision-making | It helped me understand the impact that an unplanned pregnancy would have on a teenager (Parents)  It helped me understand adolescents’ decision-making regarding pregnancy (Health professionals) |
| Encourages engagement with the situation and with Jack | It involved me in Jack's and his mother’s/father’s situation (Parents)  It made me think that I should talk more with my children about this situation (Parents)  It involved me in Jack’s situation (Health professionals)  The video helped me put myself in Jack’s shoes (Adolescents) |
| Includes appropriate questions | The questions helped me reflect on the topic presented in the video (Adolescents) |
| Suitable for addressing the topic | It made me think about issues I hadn't considered before (Parents)  I think it is an appropriate way to reflect and/or talk with others about the topic (Adolescents)  I believe it can be useful for other teenagers to reflect and/or talk about the topic (Adolescents) |
| Relevant for inclusion in the educational system | It made me reflect on the importance of including these topics in the school curriculum (Parents)  I think it is a suitable tool to include in the sex education curriculum with teenagers (Teachers)  It is good material to include in sex education in high schools and vocational schools (Adolescents) |
| Refers to the role of education and health professionals with adolescents | It made me reflect on my work with teenagers (Health professionals)  It made me think about how to approach this topic with teenagers (Health professionals)  I identified with the health professional in the video (Health professionals) |
