# Supplementary material for: Building multi-sectoral alliances to co-design and pilot a gender-transformative comprehensive sexuality education intervention for adolescents: the case of Si Yo Fuera Juan in Uruguay
Source: Reprod Health. 2026 Feb 28;23:78. doi: 10.1186/s12978-025-02257-x (PMC13072552; doi:10.1186/s12978-025-02257-x)
Supplement: Supplementary file 2 — Supplementary Material 2. [file 12978_2025_2257_MOESM2_ESM.docx]

# Appendix 1, Good Reporting of A Mixed Methods Study (GRAMMS) framework.

| **Guideline** | **Section: paragraph** |
| --- | --- |
| (1) Describe the justification for using a mixed methods approach to the research question | Method, paragraph 2 |
| (2) Describe the design in terms of the purpose, priority and sequence of methods | Method, paragraph 3 |
| (3) Describe each method in terms of sampling, data collection and analysis | Method, Participants and recruitment; Method, Data collection, Table 1; Method, Data analysis |
| (4) Describe where integration has occurred, how it has occurred and who has participated in it | Method, Data analysis, final paragraph |
| (5) Describe any limitation of one method associated with the present of the other method | Discussion, paragraph 7 |
| (6) Describe any insights gained from mixing or integrating method | Results, Stage Three, Integration of quantitative and qualitative findings. Discussion, paragraph 7. |

O'Cathain A, Murphy E, Nicholl J. The quality of mixed methods studies in health services research. J Health Serv Res Policy. 2008;13: 92-98.

# Appendix 2, Questionnaire to evaluate acceptability of the If I Were Jack intervention.

**Parents**

It helped me understand the impact that an unplanned pregnancy would have on a teenager

☐ Strongly agree  ☐ Agree  ☐ Not sure  ☐ Disagree  ☐ Strongly disagree

It made me think about issues I hadn't considered before

☐ Strongly agree  ☐ Agree  ☐ Not sure  ☐ Disagree  ☐ Strongly disagree

It involved me in Jack's and his parents’ situation

☐ Strongly agree  ☐ Agree  ☐ Not sure  ☐ Disagree  ☐ Strongly disagree

It made me think that I should talk more with my children about this situation

☐ Strongly agree  ☐ Agree  ☐ Not sure  ☐ Disagree  ☐ Strongly disagree

It made me reflect on the importance of including these topics in the school curriculum

☐ Strongly agree  ☐ Agree  ☐ Not sure  ☐ Disagree  ☐ Strongly disagree

**Adolescents**

The video helped me put myself in Jack’s shoes

☐ Strongly agree  ☐ Agree  ☐ Not sure  ☐ Disagree  ☐ Strongly disagree

The questions helped me reflect on the topic presented in the video

☐ Strongly agree  ☐ Agree  ☐ Not sure  ☐ Disagree  ☐ Strongly disagree

I think it is an appropriate way to reflect and/or talk with others about the topic

☐ Strongly agree  ☐ Agree  ☐ Not sure  ☐ Disagree  ☐ Strongly disagree

I believe it can be useful for other teenagers to reflect and/or talk about the topic

☐ Strongly agree  ☐ Agree  ☐ Not sure  ☐ Disagree  ☐ Strongly disagree

It is good material to include in sex education in high schools and vocational schools

☐ Strongly agree  ☐ Agree  ☐ Not sure  ☐ Disagree  ☐ Strongly disagree

**Health professionals**

It made me reflect on my work with teenagers

☐ Strongly agree  ☐ Agree  ☐ Not sure  ☐ Disagree  ☐ Strongly disagree

It helped me understand adolescents’ decision-making regarding pregnancy

☐ Strongly agree  ☐ Agree  ☐ Not sure  ☐ Disagree  ☐ Strongly disagree

It made me think about how to approach this topic with teenagers

☐ Strongly agree  ☐ Agree  ☐ Not sure  ☐ Disagree  ☐ Strongly disagree

It involved me in Jack’s situation

☐ Strongly agree  ☐ Agree  ☐ Not sure  ☐ Disagree  ☐ Strongly disagree

I identified with the health professional in the video

☐ Strongly agree  ☐ Agree  ☐ Not sure  ☐ Disagree  ☐ Strongly disagree

**Teachers**

It made me reflect on my work with teenagers

☐ Strongly agree  ☐ Agree  ☐ Not sure  ☐ Disagree  ☐ Strongly disagree

It helped me understand adolescents’ decision-making regarding pregnancy

☐ Strongly agree  ☐ Agree  ☐ Not sure  ☐ Disagree  ☐ Strongly disagree

It made me think about how to approach this topic with teenagers

☐ Strongly agree  ☐ Agree  ☐ Not sure  ☐ Disagree  ☐ Strongly disagree

It involved me in Jack’s situation

☐ Strongly agree  ☐ Agree  ☐ Not sure  ☐ Disagree  ☐ Strongly disagree

I think it is a suitable tool to include in the sex education curriculum with teenagers

☐ Strongly agree  ☐ Agree  ☐ Not sure  ☐ Disagree  ☐ Strongly disagree

#

# Appendix 3, Questionnaire to evaluate Si yo fuera Juan intervention for adolescents.

1. What is your gender identity?

☐ Female  ☐ Male  ☐ Gender fluid  ☐ Other

2. How often were the “If I Were Juan” activities carried out in your school?

☐ Once a week  ☐ Twice a week  ☐ Other (please specify)

3. How would you rate the presentation of “If I Were Juan” carried out by the teachers at your school?

☐ Very goog  ☐ Good  ☐ Neither good nor bad  ☐ Bad  ☐ Very bad

4. What did you think about the video about Juan and Ema? (Open-ended)

5. Of all the activities done in class as part of “Si yo fuera Juan”, which one did you like the most, and why? (Open-ended)

6. Of all the activities done in class as part of “Si yo fuera Juan” , which one did you like the least, and why? (Open-ended)

7. Were you able to participate in all the activities?

☐ Yes  ☐ No

8. Did your parent, guardian, or family member participate in the home-based activity proposed by “Si yo fuera Juan.”?

☐ Yes  ☐ No

9. I enjoyed participating in the activities of “Si yo fuera Juan.”

☐ Strongly agree  ☐ Agree  ☐ Not sure  ☐ Disagree  ☐ Strongly disagree

10. The activities of “If I Were Juan” helped me learn more about sexual health, relationships, and pregnancy.

☐ Strongly agree  ☐ Agree  ☐ Not sure  ☐ Disagree  ☐ Strongly disagree

11. The whole class enjoyed participating in the activities of “Si yo fuera Juan.”

☐ Strongly agree  ☐ Agree  ☐ Not sure  ☐ Disagree  ☐ Strongly disagree

12. The teachers made us feel comfortable while doing the activities of “Si yo fuera Juan.”

☐ Strongly agree  ☐ Agree  ☐ Not sure  ☐ Disagree  ☐ Strongly disagree

13. I think “If I Were Juan” could be very useful for other teenagers my age.

☐ Strongly agree  ☐ Agree  ☐ Not sure  ☐ Disagree  ☐ Strongly disagree

14. I believe my parents (mother, father, or other adult family member) enjoyed participating in the home-based activities proposed by “Si yo fuera Juan.”

☐ Strongly agree  ☐ Agree  ☐ Not sure  ☐ Disagree  ☐ Strongly disagree

15. If you had to rate the “Si yo fuera Juan” program from 1 to 10 (where 1 is very bad and 10 is excellent), what score would you give it? (Rating from 1 to 10)

16. Do you have any suggestions to improve the “Si yo fuera Juan” program? (Open-ended)

#

# Appendix 4, Dimensions linked to questionnaire’ items

| **Dimension name** | **Items** |
| --- | --- |
| Shows the effects of pregnancy and decision-making | It helped me understand the impact that an unplanned pregnancy would have on a teenager (Parents)  It helped me understand adolescents’ decision-making regarding pregnancy (Health professionals) |
| Encourages engagement with the situation and with Jack | It involved me in Jack's and his mother’s/father’s situation (Parents)  It made me think that I should talk more with my children about this situation (Parents)  It involved me in Jack’s situation (Health professionals)  The video helped me put myself in Jack’s shoes (Adolescents) |
| Includes appropriate questions | The questions helped me reflect on the topic presented in the video (Adolescents) |
| Suitable for addressing the topic | It made me think about issues I hadn't considered before (Parents)  I think it is an appropriate way to reflect and/or talk with others about the topic (Adolescents)  I believe it can be useful for other teenagers to reflect and/or talk about the topic (Adolescents) |
| Relevant for inclusion in the educational system | It made me reflect on the importance of including these topics in the school curriculum (Parents)  I think it is a suitable tool to include in the sex education curriculum with teenagers (Teachers)  It is good material to include in sex education in high schools and vocational schools (Adolescents) |
| Refers to the role of education and health professionals with adolescents | It made me reflect on my work with teenagers (Health professionals)  It made me think about how to approach this topic with teenagers (Health professionals)  I identified with the health professional in the video (Health professionals) |

# Appendix 5, Summary of key adaptations to the IVD from If I were Jack to Si yo fuera Juan.

| **Component** | **Original (If I Were Jack)** | **Adapted (Si yo fuera Juan)** | **Rationale** |
| --- | --- | --- | --- |
| *Protagonist name* | Jack | Juan | Short, monosyllabic, timeless, culturally neutral name |
| *Language* | English (UK/Ireland) | Rioplatense Spanish with Uruguayan colloquialisms | Natural speech patterns for identification |
| *Emotional expression of protagonists* | Restrained, mature | More visible anxiety, vulnerability, emotional intensity.  E.G: Juan calling Ema “Love” (common for romantic relationships in Uruguay), angrier reaction from Juan when learning Ema was pregnant, Ema holding a tissue in the counseling scene. | Better reflects Uruguayan adolescent emotional expression |
| *Setting* | Working-class Irish neighbourhoods | Neutral Uruguayan locations (school, big park, neighbourhood belonging a small town, community football pitch) | Socioeconomic neutrality while culturally recognizable |
| *Cultural elements* | Football | Mate, deckchairs in public spaces, videogames. | Enhance local and adolescent identification |
| *Counseling scene* | External health center with dedicated counselor | School-based counselor. Local flyers on walls. | Reflects access patterns and increases credibility |
| *Accessibility* | Subtitles | Uruguayan Sign Language | Ensure inclusion of deaf/hard-of-hearing adolescents |
| *Family reactions* | Female adult present in the kitchen, less emotional responses | Female adult present in the living room with male adult, wider range of emotions displayed | Address gender stereotypes and reflect Uruguayan emotional expression |
| *Peer interactions* | Formal language.  E.G: “We are with you through this” | Use of nicknames, emoji use in a chat resembling Whatsapp, informal communication  E.G: “You are not alone in this” as a reaction. | Authentic adolescent relationships |
| *Embedded questionnaire* | Stored locally. | Stored in a secure cloud server. | Ensure confidentiality |

# Appendix 6, Summary of key adaptations to classroom materials and implementation guidelines from If I were Jack to Si yo fuera Juan.

| **Component** | **Original (If I Were Jack)** | **Adapted (Si yo fuera Juan)** | **Rationale** |
| --- | --- | --- | --- |
| *Implementation structure* | 4 sessions of one hour each or 6 sessions of 35-45 minutes. | 6 sessions of 1.5 hours each (9 hours total); flexible adaptation possible | Aligned with Uruguay's education system schedule while maintaining adaptability for community-based settings |
| *Educational settings* | Primarily designed for formal education (secondary schools) in UK/Ireland | Explicitly co-designed for both formal settings (secondary schools, technical training programmes) AND non-formal settings (youth community centres) | Maximize reach among adolescents from socioeconomically vulnerable backgrounds with limited access to formal education |
| *Activity organization* | All mandatory activities | Core activities (essential components) + supplementary activities (optional) with colour-coded thematic labels | Allow centres to prioritize according to context and adolescent needs; enable educators to quickly identify key dimensions addressed in each activity |
| *Activities* | 14 activities.  Activity 11 “Online scavenger hunt” and 12 “Jack’s card” as different activities.  UK/Ireland cultural context, references, and examples | Grouping Activity 11 and 12.  **NEW ACTIVITIES**: Activity 4 "Initial Reactions" (dedicated discussion space immediately after IVD viewing for processing emotional reactions and first impressions); Optional Activity 15: Workshop with families (facilitated by designated teachers for parents/caregivers of participating adolescents)  **ADAPTED ACTIVITIES**: Activity 7 "If I Had to Care for a Baby" (schedule revised to reflect Uruguayan daily routines, meal times, and sleeping patterns); Activity 8 "Fiction or fact" (includes abortion legislation, Uruguay data regarding pregnancy and sexual initiation, health services); Activity 9 "Juan’s dialogues" (adapted name of the activity, social media included in the different scenarios of dialogue, language and emoji use); Activity 10 "Online scavenger hunt” (directed scavenger hunt towards quality information sites, included safety practices online, designed Juan’s card, included information about health services, SRHR, helplines, QR code to access via cellphone); Activity 11 "Controversial statements" (language adaptation); Activity 12 "Families’ activity” (questions less direct towards alternatives); Activity 13 "Practical situations about consent and contraceptives” (adapted situations, language and adolescent practices). | New activities created in response to teachers' insights during workshops (e.g., need for immediate processing time). Adapted activities localized to reflect Uruguayan contexts, routines, and adolescent practices for enhanced relevance and identification. Respond to Stage One findings about family communication challenges, family engagement in CSE, strengthen school-family interaction. |
| *Website resources.* | Available information about the intervention for teachers, parents, adolescents. | Information about intervention + resources on adolescent pregnancy, sexual violence and abuse, intimate partner violence, online relationships, and health services for parents, adolescents and teachers. | Respond to specific needs identified by teachers, EAC, and policymakers in Uruguayan context; address contemporary concerns (online relationships); address needs of parents, teachers, adolescents regarding CSE. |
| *Curriculum integration* | Adapted to UK content, designed to to fit within the Learning for Life and Work Area of Learning. | Uruguayan CSE curriculum alignment and SRH rights/services/policies specific to Uruguay, session-by-session implementation plans detailed but with room for flexibility, facilitation strategies for sensitive discussions. | Address teachers' concerns about preparation, potential family opposition, and anti-gender discourse identified in Stage One; ensure alignment with national secular educational principles |
